# Distinct mechanisms regulate *Cdx2* expression in the blastocyst and in trophoblast stem cells

**DOI:** 10.1038/srep27139

**Published:** 2016-06-03

**Authors:** Teresa Rayon, Sergio Menchero, Isabel Rollán, Inmaculada Ors, Anne Helness, Miguel Crespo, Andres Nieto, Véronique Azuara, Janet Rossant, Miguel Manzanares

**Affiliations:** 1 Centro Nacional de Investigaciones Cardiovasculares (CNIC), Melchor Fernández Almagro 3, 28029 Madrid, Spain; 2 Epigenetics and Development Group; Institute of Reproductive and Developmental Biology; Faculty of Medicine; Imperial College London; London, W12 ONN UK; 3 Program in Developmental and Stem Cell Biology, Hospital for Sick Children Research Institute, 686 Bay Street, Toronto, ON M5G 0A4, Canada; 4 Department of Molecular Genetics, University of Toronto, 1 King’s College Circle, Toronto, ON M5S 1A8, Canada

## Abstract

The first intercellular differences during mammalian embryogenesis arise in the blastocyst, producing the inner cell mass and the trophectoderm. The trophectoderm is the first extraembryonic tissue and does not contribute to the embryo proper, its differentiation instead forming tissues that sustain embryonic development. Crucial roles in extraembryonic differentiation have been identified for certain transcription factors, but a comprehensive picture of the regulation of this early specification is still lacking. Here, we investigated whether the regulatory mechanisms involved in *Cdx2* expression in the blastocyst are also utilized in the postimplantation embryo. We analyzed an enhancer that is regulated through Hippo and Notch in the blastocyst trophectoderm, unexpectedly finding that it is inactive in the extraembryonic structures at postimplantation stages. Further analysis identified other *Cdx2* regulatory elements including a stem-cell specific regulatory sequence and an element that drives reporter expression in the trophectoderm, a subset of cells in the extraembryonic region of the postimplantation embryo and in trophoblast stem cells. The cross-comparison in this study of cis-regulatory elements employed in the blastocyst, stem cell populations and the postimplantation embryo provides new insights into early mammalian development and suggests a two-step mechanism in *Cdx2* regulation.

By the time of blastocyst implantation in the uterus, the three first lineages of the embryo have been established, are committed in terms of their differentiation potential and they are no longer interconvertible. The blastocyst is initially composed of an outer epithelial monolayer of trophectoderm (TE) that covers the inner cell mass (ICM) and the fluid-filled blastocoel cavity. Soon after, the ICM separates into the epiblast and the primitive endoderm. After implantation, the epiblast remains pluripotent and will give rise to all tissues of the embryo. On the other hand, the primitive endoderm and the TE will generate all extraembryonic structures needed for embryo support and nourishment through development. The primitive endoderm forms the parietal and visceral endoderm layers of the yolk sac, whereas the TE generates the trophoblast-derived structures of the embryo: parietal trophoblast giant cells that line the implantation site, the extraembryonic and chorionic ectoderm, the ectoplacental cone, and later the various trophoblast cell types of the mature placenta^1^.

This lineage restriction is mirrored in three different stem cell populations that can be derived from the blastocyst: Embryonic Stem (ES), Trophoblast Stem (TS), and eXtraembryonic ENdoderm stem (XEN) cells. All three cell types recapitulate the lineage of their appropriate blastocyst precursor when injected into blastocysts to generate chimeras^2^. In addition, TS cells can be derived not only from the blastocyst but also from the Extraembryonic Ectoderm (ExE) up to embryonic stage (E) 8.5 of the postimplantation mouse embryo^3^, raising the question of whether TS cells reach a common state during the process of derivation, no longer resembling the tissue or stage from which they were initially derived. ES, TS and XEN cells share some features of the regulatory networks active in their lineage of origin, and serve as an *in vitro* tool for investigating how these populations are established and how the set of core transcription factors responsible for their identity is assembled. Transcription factors (TFs) are essential in cell-type specification, and their expression is determined by how they are regulated. TFs can play a deterministic role, as shown in experiments in which forced TF expression reprograms cell-type specification^4^,^5^,^6^,^7^. *Cdx2* is the core TF responsible for trophectoderm development. *Cdx2* mutants die in the blastocyst stage as the TE is not properly specified and it fails to maintain epithelial integrity^8^. Also, *Cdx2* is crucial in TS cells derivation as shown by the fact that TS cells cannot be derived from *Cdx2*-mutant blastocysts, overexpression of *Cdx2* in ES cells forces their conversion to TS cells^9^, and *Cdx2* is indispensable for TS-cell self-renewal^8^. We previously characterized a TE-specific enhancer (TEE) for *Cdx2* that faithfully recapitulates the early onset of *Cdx2* expression during preimplantation development^10^. To better understand the regulation of *Cdx2* during extraembryonic development, we analyzed TEE activity in the ExE and TS cells, unexpectedly finding that this regulatory element is not active in these instances. Further analysis of the *Cdx2* genomic region identified novel regulatory elements that drive reporter activity in TS cells and in a subset of extraembryonic tissues of the postimplantation embryo. These results reveal an early regulatory switch in *Cdx2* expression and show that different inputs are needed to drive *Cdx2* expression in the blastocyst trophectoderm and in trophoblast stem cells.

## Results

### The TE-specific enhancer for *Cdx2* is inactive in extraembryonic tissues of the postimplantation embryo

**Figure 1:**
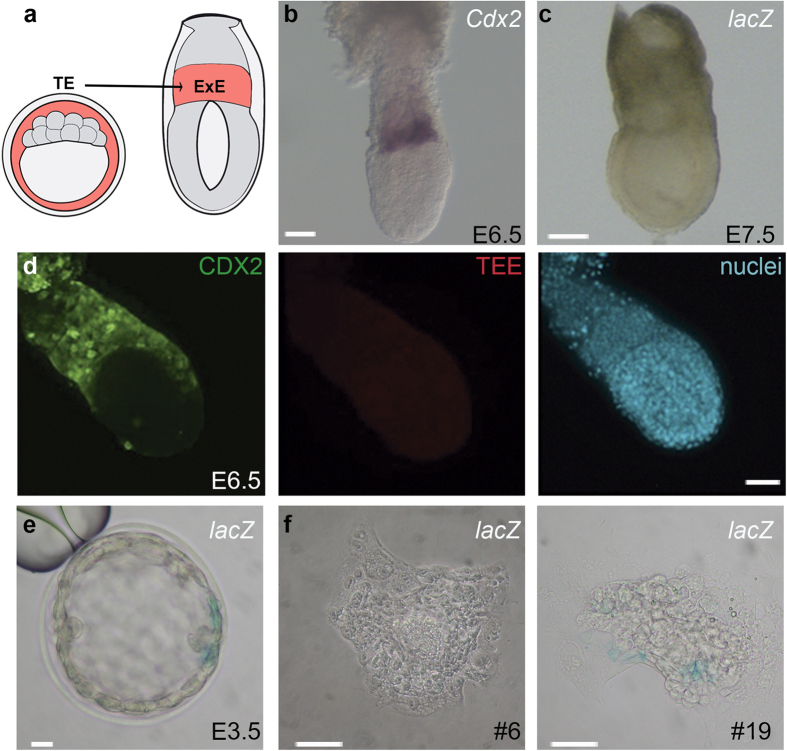
The *Cdx2* TEE is inactive in the postimplantation embryo and is progressively inactivated in blastocyst outgrowths. (**a**) Diagram of *Cdx2* expression at blastocyst and postimplantation stages (red). (**b**) Expression of *Cdx2* at E6.5 is limited to the extraembryonic ectoderm (ExE). (**c**) β-galactosidase staining in the TEE–*lacZ* reporter line at E6.5, showing lack of reporter activity. (**d**) CDX2 expression (green) and TEE activity (red) in a E7.5 embryo of the TEE-mRFP line. CDX2 and mRFP were detected by immunohistochemistry with anti-CDX2 and anti-DsRed antibodies. Nuclei are counterstained with DAPI. CDX2 is present in extraembryonic tissue, while TEE activity is absent. (**e**) β-galactosidase staining in the TEE*-lacZ* reporter line at blastocyst stage and (**f**) in two outgrowths of the same line (#6 and #19). Scale bar, 110 μm (**b**–**d**), 10 μm (**e**), and 50 μm (**f**).

We previously characterized an enhancer element (TEE) from *Cdx2* that drives reporter expression specifically in the blastocyst TE^10^. Given that *Cdx2* expression persists in the ExE of the postimplantation embryo, we examined TEE activity after the blastocyst stage in *lacZ*- and mRFP- reporter lines^10^. We expected to detect TEE mediated expression in TE derivatives that normally express *Cdx2*, such as the ExE of the early embryo at E6.5 and E7.5 (Fig. 1a,b). Surprisingly, we did not detect any reporter activity in the TEE reporter mouse lines (Fig. 1c,d), despite embryos at blastocyst stage from these same lines showed consistent and reproducible reporter expression as we have previously shown^10^. We confirmed this observation by qPCR analysis of single embryos at E3.5 and E7.5 of *Cdx2*, together with *lacZ* or *mRFP* for the TEE-lacZ and TEE-mRFP lines, respectively. We observed high expression of the reporters in transgenic blastocysts but not in the extraembryonic portion at E7.5. On the contrary, the expression of *Cdx2* in the same samples was comparable for wild type and both TEE-lacZ and TEE-mRFP embryos (Supplementary Fig. S1a).

To determine whether the TEE is switched off at the time of implantation, we cultured E3.5 blastocysts for an extra 48 h or 72 h hours in TS derivation conditions, as an *in vitro* model mirroring implantation in the uterus^11^. Under these conditions, blastocysts hatch from the zona pellucida and adhere to the dish, forming outgrowths. After 48 h in culture, embryos cultured starting from E3.5 contained 75.7% CDX2-positive cells, but after culture for a further 24 h this figure had dropped to 22.8% (Supplementary Fig. S1b,c). This is most likely due to loss of *Cdx2* expression during the differentiation of extraembryonic tissues^8^. We then cultured heterozygous blastocysts from the TEE-*lacZ* mouse line (Fig. 1e) for two days to examine reporter expression driven by the TEE upon implantation (Fig. 1f). β-galactosidase expression was detected in a few cells of just 3 out of 19 TEE-*lacZ* genotyped outgrowths after 48 h culture. Thus, in 84.21% of cases, reporter expression driven by the TEE was silenced within 2 days of outgrowth culture, despite continued robust *Cdx2* expression by the large majority of cells in the explant. These results indicate that the TEE is inactivated upon implantation, and suggests differential enhancer usage for *Cdx2* in the pre- and postimplantation embryo.

### The TE-specific *Cdx2* enhancer is inactive in trophoblast stem cells but is reactivated in blastocyst chimeras

**Figure 2:**
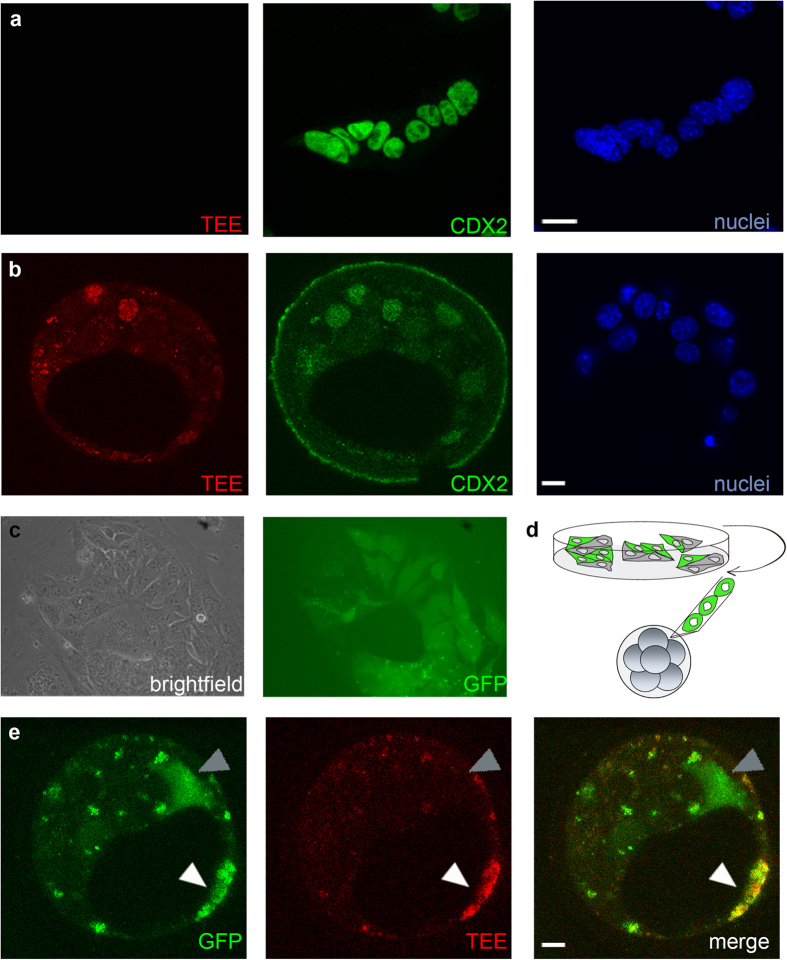
The TE-specific *Cdx2* enhancer is inactive in trophoblast stem cells but is reactivated in blastocyst chimeras. (**a**) TS_R_ cells do not show TEE-driven mRFP expression. Cells were stained for mRFP (red), CDX2 (green) and DAPI (blue). Scale bar 20 μm (**b**) TS_R_ cells re-express the TEE-driven mRFP reporter when injected into embryos. Embryos were stained for mRFP (red), CDX2 (green) and DAPI (blue). Scale bar 20 μm (**b**) GFP-infected TS_R_ cells. The left panel shows a brightfield image. The right panel shows GFP-expressing TS cells. (**d**) Injection of GFP+ TS_R_ cells into a morula. (**e**) GFP-infected TS_R_ cells re-express the TEE-driven mRFP reporter in the blastocyst. Flourescent signals from expression of GFP (green) and mRFP (red). White arrowheads point to GFP+/mRFP+ TS_R_ cells, grey arrowheads show GFP+/mRFP− TS_R_ cells. Scale bar, 10 μm.

To examine the dynamics of *Cdx2* expression in trophectoderm-derived lineages, we derived TS cells from blastocysts of mRFP- and *lacZ*- TEE reporter mouse lines (TS_R_ and TS_L_, respectively). Of 19 independent cell clones (12 TS_L_ and 7 TS_R_), 4 TS_L_ and 2 TS_R_ clones expressed comparable levels of trophectoderm pluripotency markers (*Cdx2*, *Eomes*, *Esrrb* and *Fgfr2*; Supplementary Fig. S2c) and trophoblast differentiation markers (*Stra13* and *Hand1*; Supplementary Fig. S2d) to wild type TS cells, and retained their capacity to differentiate to trophoblast giant cells upon FGF4 withdrawal (Supplementary Fig. S2a,b,e–g)^3^,^9^,^12^. The TS_L_ cells, derived from the TEE-*lacZ* mouse line, did not express TEE-driven β-galactosidase (data not shown); similarly, the TS_R_ cells did not express mRFP (Fig. 2a). Hence, TS cells do not activate the TEE, as also occurs in the ExE, and contrary to the robust TEE activity observed in the blastocyst^10^. These results suggest that TS cells are more related to ExE than to TE cells from the blastocyst regarding the regulation of *Cdx2*.

The definitive demonstration that TS cells retain multipotent stem cell features, other than their trophoblast gene expression profile and differentiation potential, is the ability to contribute to the TE lineages when injected back into the preimplantation embryo. We therefore tested if TS_R_ cells could indeed contribute to the TE and, if so, if they re-expressed mRFP directed by the TEE when located in the embryo. TS_R_ cells were injected into morula stage embryos and their contribution to the TE of the blastocyst was assessed 24 h later. Injected cells mix with host cells, resulting in the development of a mosaic embryo. Of 18 injected embryos, 8 (44.4%) contained mRFP positive cells at the blastocyst stage (Fig. 2b). All cells expressing mRFP were also positive for CDX2 protein expression, and an average of 2 CDX2-positive cells per blastocyst were mRFP+ and thus derived from the injected TS_R_ cells (Fig. 2b). In some cases we found mRFP positive cells in the subzonal space that had not integrated in the blastocyst. These cells were most likely dying, as they were not stained for DAPI.

To track TS_R_ cells in injected blastocysts, we first infected them with a lentiviral GFP construct (Fig. 2c) and sorted them immediately before injection into morulae (Fig. 2d). Of 13 injected embryos, 6 blastocysts showed GFP+ cells (46.2%). An average of 3 lentiviral-transduced TS_R_ cells were counted per blastocyst. From those, 1 or 2 injected cells re-expressed the nuclear red fluorescent reporter (Fig. 2e). The mRFP-negative TS_R_-derived cells were located outside the TE, most likely dying, and this might be why they did not properly activate the enhancer. These experiments thus show that TS_R_ cells can contribute to the TE, and can reactivate TEE-driven reporter expression when located in the appropriate cellular context.

### Chromatin landscape and regulatory activities of the *Cdx2* locus in TS cells

**Figure 3:**
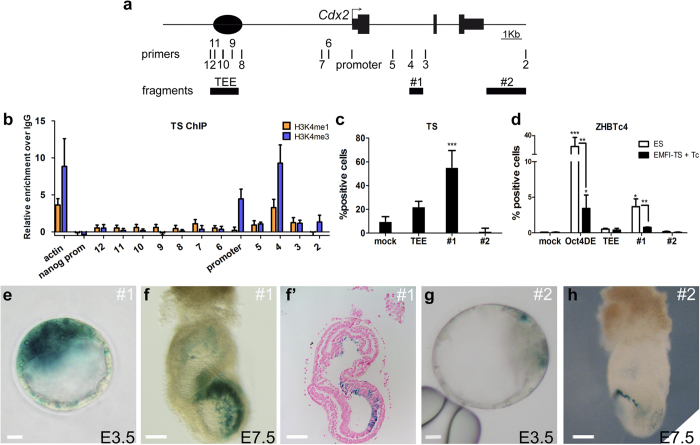
Activity of regulatory elements in the *Cdx2* locus. (**a**) Diagram of the *Cdx2* locus, showing relative positions of primers selected for ChIP-qPCR (2–12) and of the selected regulatory elements. (**b**) Relative enrichment over IgG of H3K4me1 (orange) and H3K4me3 (blue) along the *Cdx2* locus in the chromatin of TS cells; *Actin* promoter, positive control; *Nanog* promoter, negative control. Data are means ± s.e.m. n = 2. (**c**) TS transfection of different *Cdx2* regulatory fragments. Fragments were tested for regulatory capacity, and expression was compared to level obtained with empty vector (mock). Data are means ± s.e.m. n = 3. ***p < 0.001 compared with mock (Student’s t-test). (**d**) Percentage of ZHBTc4 cells showing reporter expression in transient transfections upon Tc addition. Data are means ± s.e.m. n = 3: ***p < 0.001, *p < 0.05 versus mock; **p < 0.01 between cells expressing the same fragment but maintained in ES medium versus EMFI medium + Tc (Student’s t-test). (**e**–**h**) *lacZ* reporter activity driven by fragment #1 (**e**,**f**) and fragment #2 (**g**,**h**) in transient transgenic embryos at E3.5 (**e**,**g**; scale bar 10 μm) and E7.5 (**f**,**h**). (**f’**) is a sagittal section of the embryo shown in (**f**); scale bar 110 μm.

To identify other putative regulatory elements that could drive *Cdx2* expression in TS cells and the ExE, we performed chromatin immunoprecipitation, targeting histone modifications associated with promoter and enhancer activity, and then conducted qPCR with primers spanning the *Cdx2* locus; the primers covered the TEE (amplicons 8–12), an upstream intergenic region (amplicons 6–7), the *Cdx2* promoter (promoter), the first intron (amplicons 3–5), and a downstream region (amplicon 2) shown to have enhancer activity in intestinal cells (Fig. 3a)^13^. We included the promoter of the housekeeping gene *Actin* as a positive control, and the *Nanog* promoter, which is not expressed in TS cells, as a negative control. ChIP-qPCR for H3K4me3 and H3K4me1 showed that, as expected, the *Cdx2* promoter region was enriched for H3K4me3 and depleted for H3K4me1. Consistent with the TEE being inactive in TS cells, both marks were completely absent from this region (amplicons 8–12), as well as from the upstream and downstream regions (Fig. 3b). In contrast, a region from the first intron of *Cdx2* showed enrichment for H3K4me1 and H3K4me3 (amplicon 4; Fig. 3b).

We conducted reporter assays in TS cells to test the transcriptional regulatory activity of selected fragments (Fig. 3a,c). These fragments were the 1.3 kb TEE^10^; fragment #1, a 500 bp element in the first intron of *Cdx2* that includes amplicon 4 (Fig. 3b) and has been shown to have enhancer activity in the blastocyst^14^; and fragment #2, a 1.8 kbp sequence downstream of *Cdx2* that includes amplicon 2 (Fig. 3b) and has been characterized as an enhancer driving expression in the gut caudal to the stomach^13^. Genomic fragments were cloned in the same mRFP reporter construct that was used for mouse transgenics^10^. Transfection of wild-type TS cells with the TEE showed no significant activity above that of empty vector, consistent with its lack of activity in TS_R_ and TS_L_ cells (Fig. 2a) and the absence of histone marks for enhancer activity (Fig. 3b). Transfection with fragment #2 yielded no detectable reporter expression, suggesting that it is a gut-specific enhancer and not active in extraembryonic cell types. In contrast, fragment #1 drove reporter expression in TS cells (Fig. 3c), in line with the active histone marks we identified.

To test if these regulatory regions direct reporter expression during TE lineage specification, we used the ZHBTc4 ES cell line, which transdifferentiates to a TS phenotype upon downregulation of *Oct4*. This process has been suggested to mimic the first lineage choice and the early distinction between TE and ICM. Upon addition of tetracycline (Tc), ZHBTc4 ES cells repress *Oct4*, switch off the gene regulatory pluripotency program, convert to TS cells, and upregulate *Cdx2*^9^. Immunohistochemical analysis confirmed downregulation of OCT4 and upregulation of CDX2 at 48 h after Tc administration in cells cultured in EMFI-TS medium (TS cell medium) (Supplementary Fig. S3a,b). Expression profiling of *Oct4*, *Nanog* and *Cdx2* by qPCR 48 h after Tc administration in ES culture medium detected strong reductions in *Oct4* and *Nanog*, concomitant with upregulation of *Cdx2* (Supplementary Fig. S3c). *Cdx2* upregulation was even stronger when cells were cultured in EMFI-TS medium, probably because this medium favors TS cell self-renewal and maintenance (Supplementary Fig. S3c). Next, we transfected ZHBTc4 ES cells with either empty vector, the distal enhancer of *Oct4* as positive control (*Oct4*DE)^15^, or the *Cdx2* locus fragments used in the TS cell transfection assays. *Oct4*DE directed robust expression of RFP after 48 h culture in ES medium, and fragment #1 showed comparably moderate expression (Fig. 3d). Culture in EMFI-TS medium and addition of Tc markedly reduced *Oct4*DE and fragment #1 activities. This could seem surprising, as fragment #1 is active in TS cells. However, this region contains an *Oct4* binding site^16^ that could account for its expression in ES cells as well as for its reduced expression upon OCT4 downregulation. Furthermore, it has been described that conversion of ZHBTc4 ES cells to TS-like cells is a gradual process^17^, so it is possible that this fragment is not fully active until complete transdifferentiation to TS cells has occurred. In contrast, no RFP activity could be detected in cells transfected with the TEE or fragment #2 (Fig. 3d). These results show that the TEE is not activated during conversion of ES to TS cells upon *Oct4* downregulation.

### *In vivo* activity of regulatory elements from the *Cdx2* locus

We next tested the ability of the fragments tested in TS cells to drive reporter activity *in vivo*. Fragment #1 is reported to drive ubiquitous activity at early preimplantation stages^14^, in line with our observations in ES cells and TS cells. Our results support this view, showing fragment #1-driven *lacZ* reporter expression throughout the preimplantation embryo (Fig. 3e, Supplementary Table S1). However, transient transgenesis with fragment #1 at E6.5-E7.5 only resulted in β-galactosidase expression in the posterior ectoderm and extraembryonic mesoderm of just one embryo (Fig. 3f), indicating that fragment #1 does not reproduce the *Cdx2* expression pattern in TE derivatives. Fragment #2 did not direct reporter expression in the blastocyst above background levels (Fig. 3g, Supplementary Table S1) and did not drive ExE-specific expression in the postimplantation embryo (Fig. 3h), consistent with its lack of activity in TS cells. However, we detected consistent β-galactosidase expression in the extraembryonic endoderm in two out of six transgenic embryos, reminiscent of the expression pattern of *FoxA2*^7^,^18^. These data show that none of these genomic fragments drives expression in TE-derivatives and that the TEE is the only blastocyst TE-specific *Cdx2* element identified so far.

**Figure 4:**
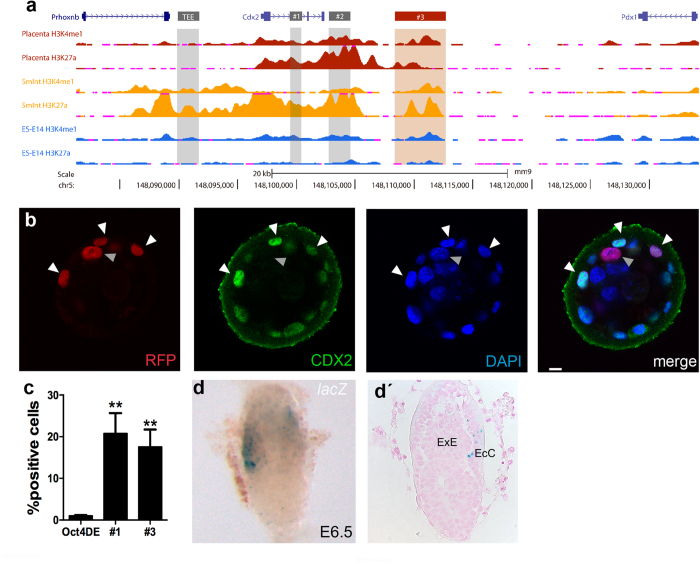
A novel regulatory element drives *Cdx2* expression in the TE, TS and a subset of extraembryonic cells. (**a**) UCSC genome browser view of the *Cdx2* region (mm9; chr5:148,081,494-148,134,458) showing (from top to bottom) ENCODE tracks of histone modifications for active enhancers (H3K4me1 and H3K27ac) in the placenta, small intestine and ES cells. The TEE and fragments #1 and #2 are highlighted in grey. Fragment #3 is highlighted in red. (**b**) mRFP (red) activity driven by fragment #3 in blastocysts, showing co-localization with CDX2 (green). Nuclei (blue) were stained with DAPI. (**c**) Fragment #3 is active in TS cells. Expression driven by fragments #1 and #3 were compared to *Oct4*DE. Data are means ± s.e.m. n = 6. **p < 0.01 (Student’s t-test). (**d**) *LacZ* reporter activity driven by fragment #3 in transient transgenic embryos. (**d’**) Sagittal section of the embryo shown in (**c)**. ExE, extraembryonic ectoderm; EcC, ectoplacental canal. Scale bar, 10 μm (**b**), 110 μm (**d**,**d’**).

To identify other regulatory elements that might drive *Cdx2* expression in TE derivatives, we examined available data on the distribution of histone modifications associated with enhancer activity (H3K4me1, H3K27ac)^19^ in placenta and small intestine, sites of *Cdx2* gene expression during development^20^, and in ES cells, where *Cdx2* is inactive. This analysis identified a region downstream of *Cdx2* that was enriched for H3K4me1 and H3K27ac in placenta and small intestine but not in ES cells (fragment #3; Fig. 4a). This fragment was active in the TE in transient transgenic blastocysts (Fig. 4b; Supplementary Table S1), as detected by co-localization with endogenous CDX2 in 75% of the mRFP positive cells. We then tested its activity in TS cells, finding that it drove reporter expression to similar levels as fragment #1 (Fig. 4c). However, when we tested if this genomic fragment was active in the ExE at postimplantation stages, we found directed reporter expression at E6.5 only in a subset of cells located in the extraembryonic portion (Fig. 4d,d’) that could correspond to the ectoplacental canal, a site of endogenous *Cdx2* expression^20^, but not throughout the ExE.

## Discussion

The first intercellular distinction in the developing embryo is produced with the formation of the trophectoderm. The actions of the core set of transcription factors responsible for establishing and maintaining the first lineages are well understood^5^,^21^,^22^, but little is known about how the expression of these factors themselves is regulated and limited to specific subpopulations of cells in the embryo. In addition, the same transcription factors operate in various contexts during embryogenesis. Thus, the identification of non-coding regulatory sequences responsible for the expression of the main transcription factors permits a better understanding of the dynamic expression profile of these genes.

In this study, we sought to identify regulatory elements implicated in different phases of *Cdx2* expression in the early stages of mouse development. The *Cdx2*-TEE enhancer is a Hippo and Notch response element that reproduces the early onset of *Cdx2* expression, when the TE is first specified^10^. To our surprise, we find that the TEE does not drive reporter activity past the blastocyst stage in TE derivatives such as the ExE, and is also inactive in TS cells. We have shown that the transcriptional effectors of the Hippo and Notch signaling pathways (TEAD4 and RBPJ, respectively) converge on the TEE to regulate *Cdx2* expression in the blastocyst^10^. A possible explanation is that although *Tead4* is expressed in the ExE of the postimplantation embryo^23^, the input from Notch is only transiently evident in a few cells of this tissue^24^. In the search for extraembryonic regulatory elements of *Cdx2*, we identified a regulatory element in the first intron of *Cdx2* that directs reporter expression in TS cells but does not direct extraembryonic expression in the embryo. This element is active not only in TS cells but also in ES cells, discarding it as an extraembryonic-specific enhancer. In addition, we characterize a novel regulatory element downstream of *Cdx2* that directs reporter expression in the blastocyst, in TS cells and in a subpopulation of the extraembryonic component of the gastrulating embryo.

Our results show that the regulation of *Cdx2* in TE and TS cells depends on different inputs and that *Cdx2* regulation is context-dependent. Strikingly, although TS cells are derived from the TE, discrete *Cdx2* cis-regulatory elements active in the TE show different ability to direct reporter expression in TS cells, and do not reproduce the postimplantation extraembryonic expression pattern of *Cdx2*.

Similar findings have been shown for the regulation of *Oct4*. In ES cells, two enhancers have been described; however, only one is able to direct reporter expression to the ICM of the blastocyst^15^,^25^. TS cells display a gene expression profile that resembles trophoblast cells in the ExE rather than the TE^6^,^12^, and some key factors of the trophoblast gene regulatory network are expressed only in the postimplantation ExE^26^. Although this suggests that TS cells share their regulation with the ExE and are distinct from the TE, we have not been able to identify any *Cdx2* enhancer that drives robust TS and ExE expression. The RNA profiles of TS cells derived at E3.5 or E6.5 cluster according to the developmental stage^27^, suggesting that additional regulatory differences may exist.

It is thus tempting to propose a two-stage regulatory model for *Cdx2*. In this model, initiation of *Cdx2* would depend strongly on the TEE, by reading Hippo and Notch inputs; in contrast, *Cdx2* extraembryonic expression upon implantation would be maintained by a combination of other elements that are active in TS cells and the postimplantation embryo. The different requirements for *Cdx2* in the blastocyst TE and in TS cells have been recently illustrated by the reprogramming of mouse embryonic fibroblasts to TS cells, a process that does not require *Cdx2*^28^,^29^. What we describe here for *Cdx2* regulation may occur in a similar fashion in other genes in the TS gene regulatory network, and it will be interesting to systematically characterize the regulatory elements unique to TS cells versus those shared with the TE or ExE lineages.

## Methods

### Construct generation for microinjection

*Cdx2* genomic regions were amplified by PCR using BAC RP245I065 as template. This BAC covers the whole intergenic region containing mouse *Cdx2* and was obtained from the BACPAC Resources Center (http://bacpac.chori.org/). Primers used for PCR, together with the lengths of corresponding amplified fragments, were as follows: TEE, CACACGGATGAATTGTCTGG and AACAGGGACAGGTGAGATGG (1329 bp); fragment #1, AGTTGGAGAGGTCTCACATCAAA and AGATTTGGTTCTGTCGTCTTCTG (563 bp), #2-LacZ, TCCATTATTCGCTCTAAAACAGC and CGAGTCACTGATCTGTGTAACGA (1842 bp), #2-mRFP, GTGTCTCTGGGTCTGAAGCT and CTGTAGGAAAGGAAGGGTGT (509 bp), #3, GTTTTGTTTTCCCGGTTGTG and TGTCTGAGATCCTGTCTTAG (4484 bp). Each fragment was subcloned and linearized as described^10^ for transgenic assays. Genomic fragments used in TS and ZHBTc4 cell transfections were subcloned in the beta-globin promoter/mRFP reporter construct.

### Embryo collection and culture

For characterization of TEE*-lacZ* and TEE-mRFP mouse lines, E0.5 embryos were collected from swollen ampulas, treated with hyaluronidase (Sigma) to remove cumulus cells and cultured until the blastocyst stage at 37.5 °C in 5% CO_2_ in air, in M16 medium (Sigma) covered with mineral oil (Sigma).

### Transient transgenic analysis

For the generation of transient transgenic embryos, F1 (C57Bl/6xCBA) females were superovulated to obtain fertilized oocytes^30^. Each construct was microinjected at 3–6 ng/μl into the pronucleus of fertilized oocytes at 0.5 dpc. Blastocysts were stained for mRFP and CDX2 and blastomeres scored for co-staining. Postimplantation embryos were collected from E6.5 to E7.5, fixed, and stained for β-galactosidase activity. Transgenic efficiency was calculated by genotyping embryos for *LacZ* (*LacZ* F, GCGACTTCCAGTTCAACATC, *LacZ* R, GATGAGTTTGGACAAACCAC) and *Myogenin* as an internal control (F, CCAAGTTGGTGTCAAAAGCC and R, CTCTCTGCTTTAAGGAGTCAG).

### Animal experimentation

Animal procedures were performed in accordance with Spanish (RD 53/2013) and European (Directive 2010/63/EU) regulations, approved by the UAM (Universidad Autónoma de Madrid) Animal Experimentation Ethics Committee, and authorized by the Regional Government of Madrid (reference CNIC15/14).

### Outgrowth formation

For trophoblast outgrowth formation assays, blastocysts were individually cultured in EMFI-TS medium in μ-Slide 8 well chambered coverslips (ibidi) pre-coated with 0.1% gelatin (Sigma)^31^. Outgrowths were cultured for 48–72 h and subsequently stained for CDX2 or β-galactosidase activity. Individual outgrowths were genotyped directly as previously described for preimplantation embryos^10^, after observation and X-Gal staining.

### Trophoblast stem cell derivation and cell culture

TS cells were derived essentially as described^3^,^32^. E3.5 blastocysts were obtained from wild-type superovulated females. For the derivation of TS_L_ cells, wild-type females were mated with TEE*-lacZ* heterozygote males. TS_R_ cells were derived from crosses of homozygote TEE-mRFP and wild-type mice. Blastocysts were plated onto 4 well plates coated with 5 × 10^4^ inactive mouse embryonic fibroblasts (EMFIs) in EMFI-TS medium supplemented with 37.5 ng/ml FGF4 and 1.5 μg/ml heparin. Plated blastocysts hatch, adhere to the well and form outgrowths on the dish. Depending on the individual blastocyst, outgrowths were harvested by trypsinization on days 4 to 6, and subsequently replated until TS cell colonies were clearly visible. After 20–21 days, MEFs were removed and TS-derived cells were cultured in EMFI-TS medium on plastic dishes. Twelve independent clones (TS_L_) were derived from the TEE*-lacZ* line and 7 TS clones (TS_R_) from the TEE-mRFP mouse line. Four TS_L_ and two TS_R_ clones were selected for further characterization. TS lines were genotyped as described^10^.

The wild type B1-TS cell line and the TS_R_ and TS_L_ cell lines were maintained as described^3^,^17^,^33^. ZHBTc4 ES cells were cultured on gelatin-coated dishes in ES medium supplemented with 1000 U/μl LIF (ESGRO-LIF; Millipore). Repression of *Oct4* (official name *Pou5f1*) in ZHBtc4 ES cells was induced by addition of tetracycline (Sigma) at 1 μg/ml in ES medium or EMFI-TS medium^9^ in the presence of 25 ng/ml FGF4 (R&D Systems) and 1 μg/ml heparin (Sigma).

### Immunohistochemistry

Immunohistochemistry on transient transgenic blastocysts was performed as previously described^34^. Cells were cultured on gelatin-coated glass coverslips and fixed in 4% paraformaldehyde in PBS. Samples were permeabilized and blocked at room temperature before incubation. The following antibodies and dilutions were used: monoclonal mouse anti-CDX2 (MU392-UC, BioGenex) 1:200, rabbit polyclonal living colours DsRed (632496 Clontech) 1:500, monoclonal mouse anti-OCT4 (sc-5279, Santa Cruz) 1:200. Nuclei were visualized by incubating embryos in 1 μg/ml DAPI, and F-actin in the cell membrane was detected by staining with a 1:300 dilution of rhodamine-phalloidin (Molecular Probes). For double staining of CDX2 and mRFP in transient transgenic blastocysts, we scored 43 mRFP positive embryos. In total, we counted 446 mRFP positive blastomeres, of which 333 were also CDX2 positive.

### Quantitative-PCR

RNA was isolated from TS and ZHBTc4 cells with the RNeasy Mini Kit (Qiagen) and then reverse transcribed using the High Capacity cDNA Reverse Transcription Kit (Applied Biosystems). RNA from single blastocysts or from the extraembryonic tissue of E7.5 embryos was isolated with the Arcturus PicoPure RNA Isolation Kit (Applied Biosystems) and reverse transcribed using the Quantitect Kit (Qiagen). TEE-mRFP blastocysts were selected for RFP expression prior to RNA isolation, and *lacZ* blastocysts were retrospectively assigned as *lacZ* positive. For E7.5 embryos, the epiblast portion was used for genotyping. cDNA was used for quantitative-PCR (qPCR) with Power SYBR® Green (Applied Biosystems) in a 7900HT Fast Real-Time PCR System (Applied Biosystems). After qPCR analysis, embryos with low amplification levels for all genes were discarded from the analysis. Expression of each gene was normalized to the expression of the housekeeping genes *Actin* and *Ywhaz*^35^. Supplementary Table S2 lists the primers used.

### Cell transfections

B1-TS cells were transfected in non-adherent dishes with 2.5 μg DNA (eGFP, empty vector, *Oct4*DE, TEE, or fragments #1, #2 and #3) using the Lipofectamine 2000 reagent (Invitrogen). Cell transfections were performed as previously described^32^ or using TS medium with 15% of KnockOut Serum Replacement (ThermoFisher) instead of serum in order to improve efficiency. ZHBTc4 cells were transfected in 6 well plates with 0.8 μg DNA (eGFP, empty vector, TEE, or fragments #1 and #2) using Lipofectamine 2000. At 6 h after transfection, medium was changed and tetracycline added when indicated. Forty-eight hours after transfection, transfected cells were counted by flow cytometry (LSRFortessa Flow Cytometer), or random fields per well were photographed (Zeiss) and fluorescent cells counted (ImageJ). Regulatory activity is expressed as the proportion of red (RFP+) cells over to total cells, normalized to transfection efficiency as measured by the number of the GFP+ transfected cells.

### Chromatin immunoprecipitation

Histone ChIP followed by quantitative PCR (qPCR) was carried out as previously described^17^. Briefly, 50 μg of precleared cross-linked chromatin from TS cells was incubated overnight with 5 μg of antibody followed by 4 h with Sepharose A beads. Eluates were digested with Proteinase K (0.5 mg/ml) and Rnase A (0.1 mg/ml), and DNA was phenol-choloroform extracted and recovered by standard precipitation with ethanol. The following antibodies were used: anti-H3K4me1 (ab8895; Abcam), anti-H3K4me3 (ab8580; Abcam), anti IgG (m7023, Sigma). Supplementary Table S2 lists the primers used for ChIP. ChIP data are represented as the fraction of the differences in ct values for each specific primer set between histone mark and IgG divided by the IgG ct value.

### Cell cycle profiling

A cell-cycle profiling assay was performed to evaluate the capacity of the selected clones to differentiate in normal TS culture conditions and upon FGF4 removal. Cell cycle was profiled by flow cytometry using an LSRFortessa Flow Cytometer. Briefly, trypsinized cells were fixed in 70% ethanol, treated with RNAse at a final concentration of 100 μg/ml, and resuspended in 0.003% propidium iodide solution. DNA content was scored in TS cells (2n/G1 phase), replicating TS cells (up to 4n/S-G2-M), and endoreduplicating cells (4n or >4n). Due to their size, some giant cells might not have been included in the flow cytometry, which was thus unable to detect all the differentiated cells.

### TS cell infection

Lentiviruses encoding the GFP reporter were generated by transient transfection of 293T cells. For transduction, 10^6^ TS cells were seeded in suspension, followed by addition of 0.25 mL vector suspension in RPMI medium (1 × 108IU/mL, MOI = 100). Eight hours post infection, vector suspension was removed and transduced cells were seeded in a 24 well dish with fresh EMFI-TS medium, and incubated at 37 °C with 5% CO_2_. Transduction efficiencies were evaluated at 48 h post-infection.

### TS cell injection

Eight to 10 TS_R_ cells were microinjected into 8-cell stage embryos using standard techniques^30^. Morulae were incubated overnight in microdrops of M16 medium (Sigma) under mineral oil. Endogenous fluorescence was assessed the next day. Immunohistochemistry for CDX2 and mRFP1 was performed as described^10^.

### Imaging

Images of transfected cells were acquired with a Zeiss Axiover 200 M inverted microscope. Nikon Eclipse 90i and Olympus BX51 microscopes were used to image histological sections. Confocal images of cells were acquired with a Leica SpE microscope (20x or 40x objective). Confocal images of microinjected or antibody-stained embryos were acquired with a Leica SP5 confocal microscope. Images were acquired with a 63× objective and 2× zoom every 2.5 μm. Images of lacZ-stained blastocysts and outgrowths were obtained with a Leica DMIRE2 inverted microscope. Images were prepared for figures using Adobe Photoshop CS5.

### Statistics

Statistical analyses were performed with GraphPad Prism 5. Data are presented as means ± s.e.m or ± s.d. as indicated in the figure legends. Differences were considered statistically significant at p < 0.05. p values were calculated by Student’s t-test for comparisons of two groups, and by ANOVA with Bonferroni post-test for multiple pair-wise comparisons.

## Additional Information

**How to cite this article**: Rayon, T. *et al.* Distinct mechanisms regulate *Cdx2* expression in the blastocyst and in trophoblast stem cells. *Sci. Rep.* **6**, 27139; doi: 10.1038/srep27139 (2016).

## Supplementary Material

Supplementary Information
