# Supplementary material for: Distinct mechanisms regulate Cdx2 expression in the blastocyst and in trophoblast stem cells
Source: Sci Rep. 2016 Jun 3;6:27139. doi: 10.1038/srep27139 (PMC4891713; doi:10.1038/srep27139)
Supplement: Supplementary Information [file srep27139-s1.pdf]

Supplementary information

Distinct mechanisms regulate Cdx2 expression in the blastocyst and in trophoblast stem cells

Teresa Rayon, Sergio Menchero, Isabel Rollán, Inmaculada Ors, Anne Helness, Miguel Crespo, Andres Nieto, Véronique Azuara, Janet Rossant and Miguel Manzanares.

The following figures and tables are included as Supplementary Information

Supplementary Figure S1. Characterization of reporter expression in TEE transgenic lines and of the trophoblast outgrowth model.

Supplementary Figure S2. Characterization of TS_L and TS_R cells.

Supplementary Figure S3. ZHBTc4 cell line characterization.

Supplementary Table S1. Table with results of transgenic assays.

Supplementary Table S2. List of qPCR primers for RNA expression and for ChIP-qPCR experiments.

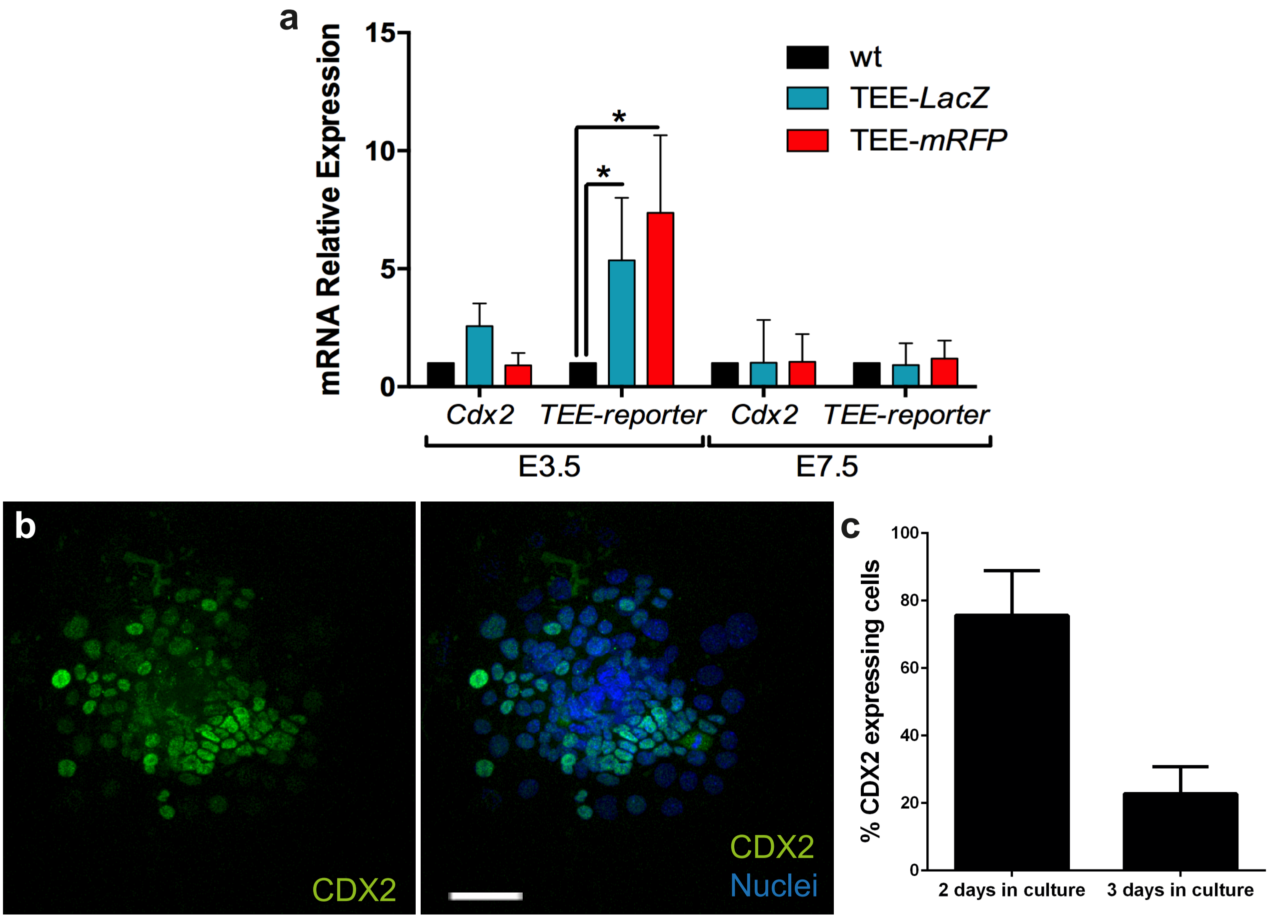

Supplementary Figure S1. Characterization of reporter expression in TEE transgenic lines and of the trophoblast outgrowth model.

(a) mRNA relative expression of *Cdx2*, *LacZ* and *mRFP* in E3.5 and the ExE of E7.5 TEE-LacZ and TEE-mRFP embryos, respectively. Data are means ± s.e.m. n=3. *p<0.05 compared with wild-type sibling embryos (Student's t-test). (b)Immunostaining for CDX2 (green) of an outgrowth cultured for two days. Nuclei are counterstained with DAPI. Scale bar 50μm. (c) Percentage of CDX2 expressing cells in outgrowths grown for two or three days.

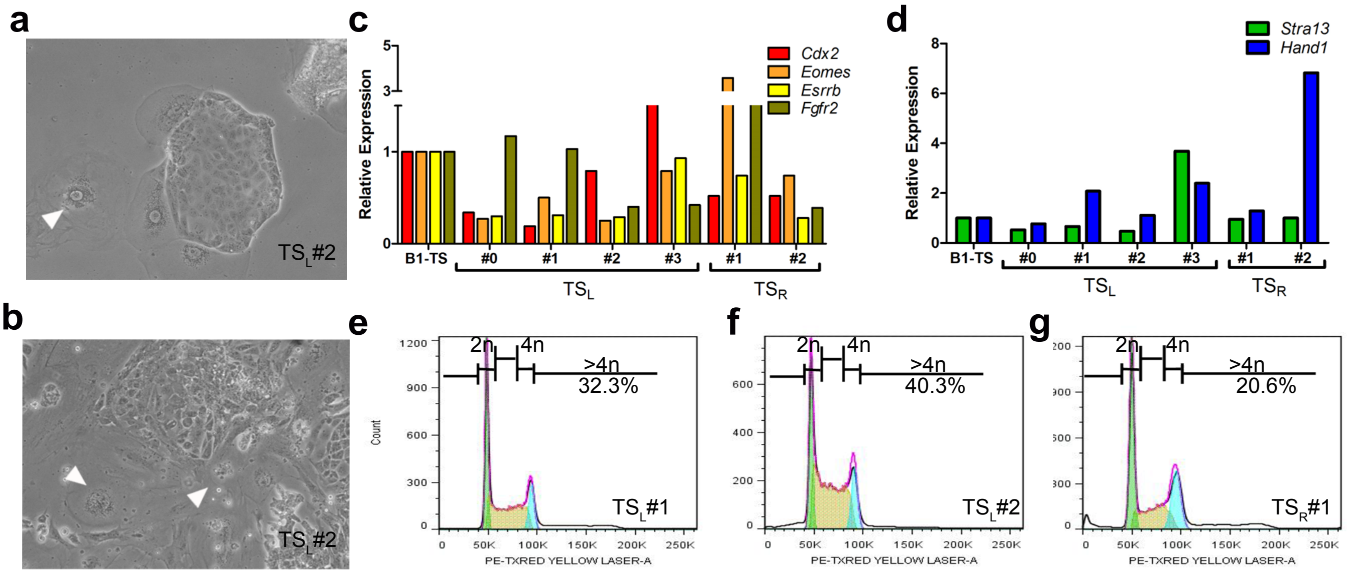

Supplementary Figure S2. Characterization of TS_L and TS_R cells.
(a) Representative TS_L epithelial colony in early passages. (b) TS_L clone upon FGF4 removal subjected to differentiation. Arrowheads point to differentiated cells. (c-d) Relative expression of (c) TS pluripotency and (d) differentiation markers in TS_L (#0-#3) and TS_R (#1- #2) clones. TS_L #0 was genotyped as *lacZ* negative. (e-g) DNA content in different (e, f) TS_L and (g) TS_R clones. DNA content is represented by the propidium iodide intensity.

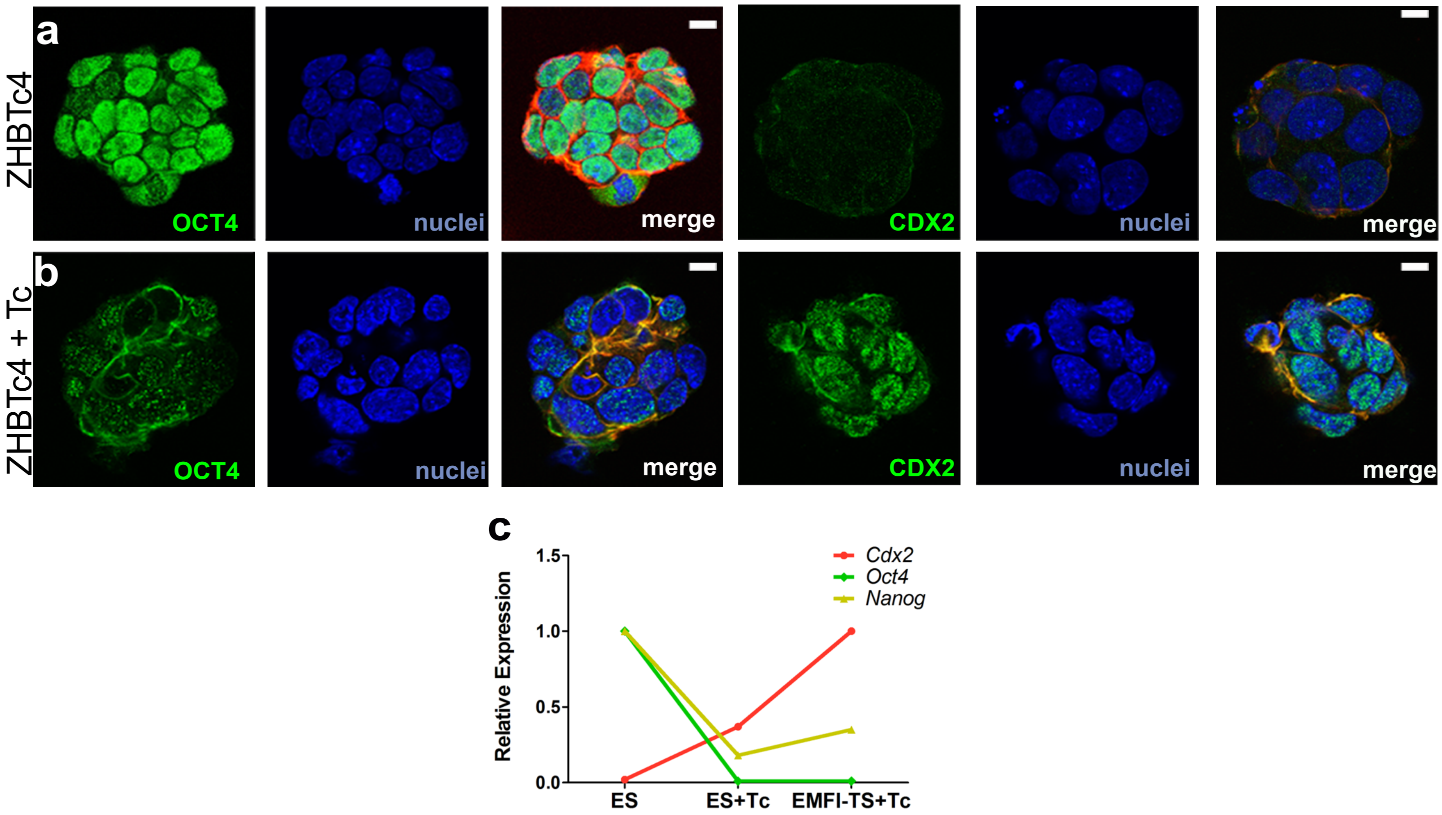

Supplementary Figure S3. ZHBTc4 cell line characterization.

(a, b) OCT4 (green) and CDX2 (green) expression in ZHBTc4 cell line in (a) ES medium or (b) upon tetracycline induction in EMFI-TS medium (Tc, 48h). Nuclei (blue) were stained with DAPI and F-actin with Rhodamine-phalloidin (red) to detect cell membrane. Scale bars, 20mm (c) Relative expression of Cdx2, Oct4 and Nanog in the ZHBTc4 cell line in different culture conditions with and without Tc.

SUPPLEMENTARY TABLE S1

		stage					
		E3.5			E7.5		
fragment	size (kb)	n reporter +	n embryos*	% reporter+	n reporter +	n tg**	% reporter+
#1	0.5	33	175	18.9%	1	11	9.1%
#2	1.8	8	73	10%	2	6	33.3%
#3	4.4	54	126	42.9%	5	16	31.2%

* includes all embryos reaching the blastocyst stage after microinjection

** includes all transgenic embryos idenitfied by PCR genotyping

SUPPLEMENTARY TABLE S2

RT-qPCR primers

Gene	forward primer	reverse primer
Actin	CAGAAGGAGATTACTGCTCTGGCT	TACTCCTGCTTGCTGATCCACAT
Cdx2	TCAACCTCGCCACAACCTTCCC	TGGCTCAGCCTGGGATTGCT
Eomes	TTCACCTTCTCAGAGACACAGTTCAT	GAGTTAACCTGTCATTTTCTGAAGCC
Esrrb	GGACACACTGCTTTGAAGCA	ACAGATGTCTCTCATCTGGC
Fgfr2	GAGGAATACTTGGATCTACC	CTGGTGCTGTCCTGTTTGGG
Gata3	GGGTTCGGATGTAAGTCGAG	CCACAGTGGGGTAGAGGTTG
Hand1	TGCACAAGCAGGTGACCCCG	CCCTTTAATCCTCTTCTCGCCG
Nanog	CTTACAAGGGTCTGCTACTGAGATGC	TGCTTCCTGGCAAGGACCTT
Oct4	ATCAGCTTGGGCTAGAGAAGGATG	AAAGGTGTCCCTGTAGCCTCATAC
Stra13	GGTGAGCAGACTACTCCATTT	GTGCCCCACATATTCCCCAC
Ywhaz	CGTTGTAGGAGCCCGTAGGTCAT	TCTGGTTGCGAAGCATTGGG
mRFP-1	GCAGAAGAAGACCATGGGCT	TGTCCAGCTTGATGTCGGTC
LacZ	TTCAACATCAGCCGCTACAG	CGTCGATATTCAGCCATGTG

ChIP –qPCR primers

Region		forward primer	reverse primer
Actin promoter		CCCCAACACACCTAGCAAAT	ACTGCCCCATTCAATGTCTC
Nanog promoter		CCCAGGTTTCCCAATGTGAAG	AAAGAGTCAGACCTTGCTGCCA
Cdx2 promoter		CTCGACGTCTCCAGCCATTGGT	CCAGCGGCCTTACGTGATTAAC
fragment #2	2	CTGCCTGCCTCCTCCCTCCA	GGGCCCCCTCTGCCTACACT
fragment #1	3	GGAACGCGTCTCACCTGCCC	CCAGCAGCCCCGCGCTATTT
	4	CCCGCGCCTGCTTTGGAAGT	GCCAGCGCGTGGTGCTCTAA
	5	CACTCCGGCAGCATTGCCCA	TGGCACAGCCAGGCCACATG
intergenic	6	GGTGGCTTGTAGAGCTGCGGT	GGGGGCGCAACCTGGAGGTA
	7	CCCAATCTCATCAAGCTGCCTTTG	TGGAACCCTACAGGAGAACCTTTG
TEE	8	AGGTTCTCCACTTGCTGCGGC	GCATCCAAGCACGGAAGTGAACA
	9	GCCCCATTCACAGTCTCCAGTTACA	TGCTTCGTTCCTCACCTTCCCCA
	10	TCCCACCGAACGCAAAACAGCT	ACCGCTCCTGTGGCCCAGAA
	11	CTCGGAGGGATAAGCTCTCAAGTGT	TGCCTCTCTGGAACAACCCGGT
	12	AGGGCTGGCATCCTCGAGCA	TGTGCCACAGCTTTTGGGCT
